# Collaborative regulation of CO_2_ transport and fixation during succinate production in *Escherichia coli*

**DOI:** 10.1038/srep17321

**Published:** 2015-12-02

**Authors:** Li-Wen Zhu, Lei Zhang, Li-Na Wei, Hong-Mei Li, Zhan-Peng Yuan, Tao Chen, Ya-Ling Tang, Xin-Hua Liang, Ya-Jie Tang

**Affiliations:** 1 Key Laboratory of Fermentation Engineering (Ministry of Education) and Hubei Key Laboratory of Industrial Microbiology, Hubei University of Technology, Wuhan 430068 China; 2 Key Laboratory of Systems Bioengineering (Ministry of Education), School of Chemical Engineering and Technology, Tianjin University, Tianjin 300072 China; 3 State Key Laboratory of Oral Diseases, West China Hospital of Stomatology, Sichuan University, Chengdu 610041 China

## Abstract

In *Escherichia coli*, succinic acid is synthesized by CO_2_ fixation-based carboxylation of C3 metabolites. A two-step process is involved in CO_2_ integration: CO_2_ uptake into the cell and CO_2_ fixation by carboxylation enzymes. The phosphoenolpyruvate (PEP) carboxylase (PPC) and carboxykinase (PCK) are two important carboxylation enzymes within the succinate synthetic pathway, while SbtA and BicA are two important bicarbonate transporters. In this study, we employed a dual expression system, in which genes regulating both CO_2_ uptake and fixation were co-overexpressed, or overexpressed individually to improve succinate biosynthesis. Active CO_2_ uptake was observed by the expression of SbtA or/and BicA, but the succinate biosynthesis was decreased. The succinate production was significantly increased only when a CO_2_ fixation gene (*ppc* or *pck*) and a CO_2_ transport gene (*sbtA* or *bicA*) were co-expressed. Co-expression of *pck* and *sbtA* provided the best succinate production among all the strains. The highest succinate production of 73.4 g L^−1^ was 13.3%, 66.4% or 15.0% higher than that obtained with the expression of PCK, SbtA alone, or with empty plasmids, respectively. We believe that combined regulation of CO_2_ transport and fixation is critical for succinate production. Imbalanced gene expression may disturb the cellular metabolism and succinate production.

Succinic acid is a dicarboxylic acid produced as an intermediate of the tricarboxylic acid (TCA) cycle, and also as one of the fermentation products of anaerobic metabolism. It has also numerous applications in agricultural, food, and pharmaceutical industries^1^. It is classified as the most promising chemical among the 12 bio-based chemicals, by the US Department of Energy^2^.

Succinic acid is produced chemically via hydrogenation of maleic acid, or through fermentation of glucose from renewable feedstock. Recent studies have shown that *Escherichia coli* is another promising mean for succinic acid production, because the bacterium can be genetically engineered with relative ease, and has the advantage of fast growth^3^,^4^,^5^,^6^.

In *E. coli*, succinic acid is synthesized by CO_2_ fixation-based carboxylation of C3 metabolites. One of the most important C3 metabolites is phosphoenolpyruvate (PEP). PEP can be converted to oxaloacetic acid (OAA) by either PEP carboxylase (PPC) or PEP carboxykinase (PCK)^7^. And then OAA is further converted to succinate through malate dehydrogenase, fumarase, and fumarate reductase. Previous studies have demonstrated that overexpression of genes related to CO_2_ fixation, such as PPC^8^, PCK^9^ and pyruvate carboxylase (PYC)^10^, increases succinate production efficiently in *E. coli*. Because the PCK activity is subject to glucose catabolite repression in *E. coli*^11^, PPC is recognized as the primary enzyme for fermentative production of succinate^12^. Overexpression of *ppc* gene from *Sorghum vulgare* in *E. coli* strain SB2020 increased succinate production by 1.5 folds^13^.

Another critical step in succinic acid production is the CO_2_ uptake by cells. In *E. coli*, the active substrate of PPC is the bicarbonate anion HCO_3_^− 14^. CO_2_ crosses the cell membrane into the cytoplasm by passive diffusion, and is converted into HCO_3_^− 14^. The slow and passive diffusion of CO_2_ into cells is a limiting step for enhancing succinic acid production. Recently, several strategies were developed through increasing the concentration of CO_2_ in the fermentation broth^14^,^15^ or accelerating the intracellular conversion of dissolved CO_2_ into bicarbonate to improve the supply of HCO_3_^−^, in order to enhance succinate production^16^. However, no literature has been found to improve succinate biosynthesis by directly enhancing HCO_3_^−^ transmembrane transport in *E. coli*.

Several HCO_3_^−^ active transporters have been discovered in cyanobacteria^17^,^18^. These HCO_3_^−^ transporters actively transport HCO_3_^−^ into cells, resulting in accumulation of HCO_3_^−^ inside the cell. Two of the efficient transporters are represented by SbtA and BicA^19^. The Na^+^-dependent SbtA transporter was originally identified in the cyanobacterium, *Synechocystis* PCC6803. It is a single gene transporter with relatively high affinity for HCO_3_^−^, requiring Na^+^ for maximal HCO_3_^−^ uptake activity^18^. The BicA transporter is also Na^+^-dependent and unrelated to SbtA. It has a relatively low transport affinity but high flux rate^19^.

In an attempt to further enhance succinic acid production, we employed a dual expression system, in which genes regulating both PEP carboxylation and CO_2_ uptake were overexpressed individually or co-overexpressed. Our results showed that the best succinate production was attained only when one CO_2_ transport and one CO_2_ fixation gene were co-expressed. This work provides useful information for metabolic regulation of CO_2_ to improve succinate production.

## Materials and Methods

### Strains and plasmids

Strains and plasmids used in this study were summarized in Table 1. Primers were summarized in Table 2. *E. coli* strain DH5α was used for plasmid construction. Strain AFP111 was kindly provided by Prof. Clark, Southern Illinois University^20^. *Synechocystis* PCC6803 was provided by Prof. Xu, Institute of Hydrobiology, Chinese Academy of Sciences^21^ and used as the *sbtA*, *bicA* and *ppc* gene donor. *Bacillus thuringiensis* BMB171 was provided by Prof. Sun, Huazhong Agricultural University^22^ and used as the *pck* gene donor. Plasmids pTrc99A and pACYC184 were used as the foundation plasmids for construction and overexpression.

### Plasmid construction procedure

The *sbtA* was amplified from *Synechocystis* PCC6803 genome by polymerase chain reaction (PCR). All PCRs were carried out based on the manufacturer’s recommended conditions (Bio-Rad, USA). The forward and reverse primers is SbtA-SacI-H and SbtA-B-His, respectively (Table 2). The PCR product was digested with SacI and BamHI and ligated into the plasmid pTrc99A. The ligated, ampicillin (Amp) resistant vector was designated as pTrc-*sbtA.* The *bicA* was amplified from *Synechocystis* PCC6803 genome by PCR with primer BicA- EcoRI-H and BicA-B-His (Table 2) and was digested with EcoRI and BamHI, and then ligated into the plasmid pTrc99A (designated as pTrc-*bicA*). The *trc-sbtA* was amplified from pTrc-*sbtA* by PCR with primers P-trc-XbaI and SbtA-SalI-His and digested with XbaI and SalI and then ligated into the plasmid pTrc-*bicA* (designated as pTrc-*bicA-sbtA*). The *ppc* gene was amplified from *Synechocystis* PCC6803 genome by PCR with primers *ppc*-EcoRI and *ppc*-BamHI, digested with EcoRI and BamHI and then ligated into the plasmid pTrc99A (designated as pTrc-*ppc*). The *trc-sbtA* was digested with XbaI and SalI and then ligated into the plasmid pTrc-*ppc* (designated as pTrc-*ppc-sbtA*). The *bicA* was amplified by PCR with primers BamHI-SD-BicA and BicA-XbaI, digested with XbaI and BamHI and then ligated into the plasmid pTrc-*ppc* (designated as pTrc-*ppc-bicA*). The *trc-sbtA* was digested with XbaI and SalI and then ligated into the plasmid pTrc-*ppc-sbtA* (designated as pTrc-*ppc-bicA-sbtA*). The *pck* was amplified from *Bacillus thuringiensis* BMB171 genome by PCR with primers *pck*-SacI and *pck*-HindIII. The PCR product was digested with SacI and HindIII and then inserted into the plasmid pTrc99a yielding the recombinant plasmid pTrc-*pck*. To construct plasmid pACYC-*trc-pck*, the *pck* expression cassette with promoter *trc* from plasmids pTrc-*pck* was digested with DrdI and BclI, and then ligated into the plasmid pACYC184 yielding the plasmid pACYC-*trc-pck.* All plasmids were introduced into *E. coli* AFP111 strain by chemical transformation. The colonies were screened by PCR amplification and confirmed for cloning accuracy by DNA sequence analysis. The transformants were designated as Tang1501 to Tang1518 (Table 1).

### Expression and detection of membrane protein

Cells of *E. coli* AFP111 transformed with various plasmids were grown in LB medium (10 g L^−1^ tryptone, 5 g L^−1^ yeast extract, and 5 g L^−1^ NaCl) at 37 °C to OD_600_ = 1.0. Gene overexpression was induced by addition of 10 μM isopropyl-β-D- thiogalactopyranoside (IPTG) (Biosharp) and grew overnight. Cells were centrifuged at 4,600 × g for 15 min and pellets were resuspended in phosphate-buffered saline (PBS) (pH 7.4). Cells were sonicated on ice for 15 min (a working period of 5 s in a 7-s interval for each cycle) at a power output of 200 W by an ultrasonic disruptor (J92-II, Xinzhi, Ningbo, China). Unbroken cells were removed by centrifugation at 10,000 × g for 15 min. Supernatant was further centrifuged at 100,000 × g for 60 min. Finally, pellets (membranes) were resuspended in 100 mM Tris buffer (pH 6.8) (ANGUS), 10% β-mercaptoethanol (AMRESCO), 4% Sodium dodecyl sulfate (SDS) (Biosharp) and stored at −80 °C.

The membrane proteins (SbtA and BicA) isolated from cells were fractionated through 10% SDS-polyacrylamide gel and electrotransferred to a polyvinylidene fluoride membrane for Western blot analysis. The membrane was incubated at room temperature for 2 h with a mouse His-tag monoclonal antibody (Jackson, USA) at a dilution of 1:2000, rinsed, and then incubated with alkaline phosphatase (AP) labeled goat anti-mouse IgG secondary antibody (Jackson, USA) at room temperature for 2 h at a dilution of 1:2000.

### HCO_3_^−^ transport activity

HCO_3_^−^ transport was determined by radioactive NaH^14^CO_3_^23^. Gene overexpression was induced by addition of 10 μM IPTG and cultured overnight. Cells were centrifuged at 4,600 × g for 15 min and pellets were resuspended in fresh fermentation medium (pH 7.0) (Composition of medium was listed in section 2.6) to OD_600_ = 10.0. A stock solution of radioactive NaH^14^CO_3_ (China Isotope and Radiation Corporation) in NaHCO_3_ (5 mM, 1.0 μCi μL^−1^) was added to cells at a final concentration of 0.185 mM. Cells were mixed and 50 μL aliquots were transferred to centrifuge tubes and incubated at 37 °C. Bicarbonate uptake was stopped by adding 1 mL non-radioactive NaH^12^CO_3_ (0.5 M). The cells were collected through filter membrane (0.45-μm, Jinteng, China) and the radioactivity was determined in a scintillation counter (Perkin Elmer, USA).

### RT-qPCR

Cells of *E. coli* AFP111 transformed with plasmids were collected at 14 h during the dual-phase fed-batch fermentation. The total RNA was extracted with Bacterial RNA Kit (Omega). The total RNA fragments were reverse-transcribed into cDNA by using PrimeScript^TM^ RT reagent Kit (Takara). 16 S rRNA was selected as the endogenous control. All cDNA samples were diluted to a final concentration of 10 ng/μL. Two-Step RT-PCR Kit with SYBR green was used with a thermal cycler (iCycler, Bio-Rad) for RT-qPCR. Primers were used at a final concentration of 0.2 μM, and 10 ng of cDNA was used as template in each 20 μL reaction. The threshold cycles for each sample were calculated from the fluorescence data with proprietary software (Bio-Rad). The fold changes for comparing the relative gene expression levels to those of the controls in the different tissues and at the different developmental stages were determined using the 2^−ΔΔCt^ method. We defined a threshold value, i.e. increases greater than 2-fold in the amount of transcripts relative to empty plasmids control samples were considered significant.

### Measurements of enzyme activity

Crude extracts for all enzyme assays were prepared by harvesting 10 mL of the cell culture from the reactor by centrifugation at 4,600 × g and 4 ^°^C for 10 min. After resuspending the cell pellets with 100 mM Tris-HCl, (pH 7.4), cells were sonicated on ice for 8 min (a working period of 8 s in a 3-s interval for each cycle) at a power output of 200 W by an ultrasonic disruptor (J92-II, Xinzhi, Ningbo, China). Cell debris was removed by centrifugation at 10,000 × g for 20 min at 4 °C. The supernatant was further centrifuged at 10,000 × g for 10 min and the resulting supernatant was used to assay enzyme activity. The PEP carboxylase (PPC) and PEP carboxykinase (PCK) activities were assayed by measuring the changes of NADH using absorbance at 340 nm^24^. PPC was monitored in a 100 μL reaction mixture containing: 66 mM Tris-HCl (pH 9.0), 10 mM MgCl_2_, 10 mM NaHCO_3_, 0.15 mM NADH (Biosharp), 0.4 U malate dehydrogenase (Amresco), and 10 μL cell extract. The PCK activity was determined in a 100 μL mixture containing: 100 mM Tris-HCl (pH 7.8), 75 mM NaHCO_3_, 16 mM MgCl_2_, 10 mM ADP (Biosharp), 0.2 mM NADH, 0.4 U malate dehydrogenase, and 10 μl cell extract^24^. The mixture was incubated for 15 min at 37 °C to activate PPC or PCK, after which the reaction was started by the addition of 5 mM PEP. 1 U of PPC or PCK activity was defined as the amount of enzyme needed to oxidize 1 μM NADH per min at room temperature. The total protein concentration in crude cell extract was measured by Bradford’s method^25^ with bovine serum albumin as a standard. Enzyme assays were performed in triplicate, and if the discrepancy was greater than 10%, another pair of assays was performed.

### Fed-batch culture

During strain construction, cells of *E. coli* AFP111 were grown aerobically at 37 ^°^C in LB medium. Preculture and fermentation medium consisted of the following components (g L^−1^): glucose, 35; yeast extract, 10; tryptone, 20; K_2_ HPO_4_·3 H_2_O, 0.90; KH_2_PO_4_, 1.14; (NH_4_)_2_SO_4_, 3.0; MgSO_4_·7 H_2_O, 0.30 and CaCl_2_·2 H_2_O, 0.25. Antibiotics were included as necessary at the following concentrations: 100 μg mL^−1^ ampicillin, 50 μg mL^−1^ kanamycin, and 10 μg mL^−1^ chloroamphenicol. Protein expression was induced by the addition of IPTG to a final concentration of 10 μM.

The first pre-culture medium (50 mL) was prepared in a 250-mL flask, and a colony from a plate culture was inoculated and incubated for 12 h at 37 °C on a rotary shaker at 250 rpm. For the second pre-culture, 50 mL of pre-culture medium was prepared in a 250-mL flask, inoculated with 100 μL of the first pre-culture broth and incubated for 12 h at 37 °C on a rotary shaker at 250 rpm.

Dual-phase fed-batch fermentation was conducted with 5 L of initial fermentation medium in a 7.5 L Bioflo 115 fermenter (New Brunswick Scientific). A 5% (v v^−1^) inoculum was used from the second preculture. At the beginning of the aerobic growth phase, 35 g L^−1^ glucose was added. During growth, oxygen-enriched air (DA-5001, Dynamic, China) was sparged at 0.1–0.4 vvm under agitation of 300–800 rpm to maintain the dissolved oxygen (DO) above 40%. When its concentration dropped below 1 g L^−1^, the aerobic growth phase was terminated by switching the inlet gas composition to oxygen-free CO_2_ at 0.2 mL min^−1^. The pH was controlled at 7.0 with 20 g L^−1^ MgCO_3_ and 5 M NaOH. Agitation was reduced to 400 rpm and initial glucose was maintained at 40 g L^−1^. When the residual sugar concentration dropped below 10 g L^−1^, a concentrated sterile glucose solution (800 g L^−1^) was fed into the media to maintain the residual glucose concentration around 40 g L^−1^.

Determination of cell mass and measurements of residual sugar and succinate concentration were performed as previously reported^26^.

Six cultures were carried out simultaneously in stirred-tank bioreactors with different engineered strains under identical experimental conditions, which ensured accurate head-to-head comparisons. The results presented here were reproducible in another experiment (data not shown).

### Succinate determination

For succinate determination, 1 mL of methanol and 1 mL of acetonitrile were added to 1 mL of fermentation broth to remove proteins, and the sample was incubated at 4 °C overnight. After centrifugation at 10,800 × g for 30 min, the supernatants were filtered through a 0.22-μm filter and analyzed by high-performance Dionex Ultimate 3000 liquid chromatographer (Thermo Scientific) using a Reprosil-Pur Basic C18 column. The optimized mobile phase was 5 mM KH_2_PO_4_ water solution, with pH adjusted to 2.8 by H_3_PO_4_. The column oven temperature was maintained at 40 °C, and the flow rate was maintained at 1 mL min^−1^. The detection wave was 210 nm.

### Data analyses

All experiments were performed in triplicate. Data were expressed as means ± standard deviations, and they were analyzed using SPSS 19.0 for Windows software. One-way analysis of variance was performed. Scheffe multiple comparison procedure (alpha ≤ 0.05) was used for individual variables to compare means and to assess significant differences.

## Results and Discussion

### Individual regulation of CO_2_ fixation or transport

#### Effect of PPC and PCK on succinate production

PEP carboxylation is one of the rate-limiting reactions in succinate production^27^. To improve succinate production, *ppc* from *Synechocystis* PCC6803 and *pck* from *B. thuringiensis* BMB171 were overexpressed individually or in combination.

**Figure 1:**
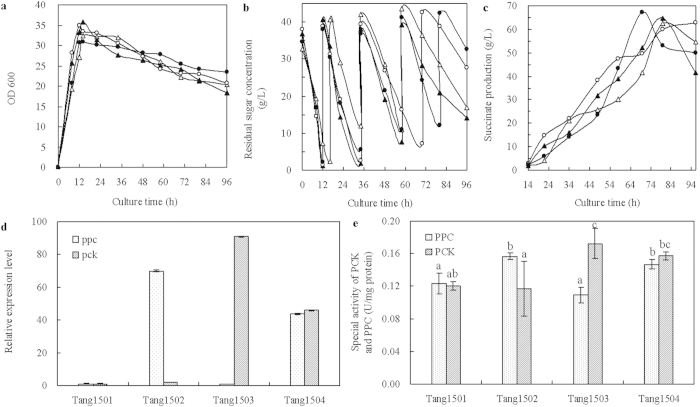
Effect of CO_2_ fixation genes expression on the cell growth (**a**), glucose consumption (**b**), the succinate production (**c**), relative expression levels of *ppc* and *pck* (**d**), and the enzyme activities of the PCK and PPC (e) in fed-batch fermentation. Symbols for *E. coli* strains: Tang1501 (pACYC184) (open triangle, 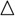), Tang1502 (*ppc*) (black triangle, 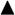), Tang1503 (*pck*) (open circle, ○), Tang1504 (*ppc* and *pck*) (black circle, 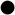). Error bars show standard deviation (n = 3). Different letters (e.g., **a–**c) were assigned to significantly different groups, and for the results between two groups, a combination of the two corresponding letters was used (e.g., **a,b**).

As shown in Fig. 1a–c, overexpression of *ppc* or/and *pck* apparently failed to affect cell growth, pattern of glucose consumption and succinate biosynthesis significantly. The succinate production obtained with Tang1501 (pACYC184), Tang1502 (*ppc*), Tang1503 (*pck*), and Tang1504 (*ppc* and *pck*) was between 62.6 and 67.3 g L^−1^. The concentrations of succinate were decreased after 70 or 80 h. Although carbon source feeding was performed, nitrogen sources, inorganic salts and vitamins may be insufficient at the end of the fermentation. Lack of nutrients may limit cellular activity and metabolic efficiency. There was a similar phenomenon could be observed in the previous report^28^.

The RT-qPCR analysis indicated that *ppc* and *pck* was overexpressed. Although the expression of *ppc* and *pck* exhibited 43.7- to 90.9-fold higher levels compared with that of empty plasmid control (Fig. 1d), and the activity of PPC and PCK was significantly improved by individual or combined expression of *ppc* and *pck* genes (Fig. 1e). The overexpression of PPC and/or PCK showed insignificantly improved effect on the succinate biosynthesis (Fig. 1c). It was probably due to low substrate supply. As the substrate for carboxylation enzyme, the diffusion of HCO_3_^−^ through the cell membrane was the key limiting process for succinate formation^14^. Permeation of HCO_3_^−^ through the lipid membrane is insignificant. Therefore, the speed and flux of substrate supply might be limited by the passive diffusion transportation mode of HCO_3_^−^. Although the activity of carboxylation enzyme was increased, it was difficult to improve the carboxylation reaction flux due to insufficient HCO_3_^−^.

#### Overexpression of BicA or SbtA significantly increases HCO_3_^−^ uptake but decreases succinate production

In order to increase the HCO_3_^−^ uptake, two heterogeneous HCO_3_^−^ transport genes of *Synechocystis* PCC6803, *bicA* and *sbtA* were overexpressed in *E. coli* AFP111 cells. The BicA and SbtA were chosen for their highly conserved adaptability for CO_2_ assimilation, and for the relative ease of genetic manipulation compared with other transporters.

**Figure 2:**
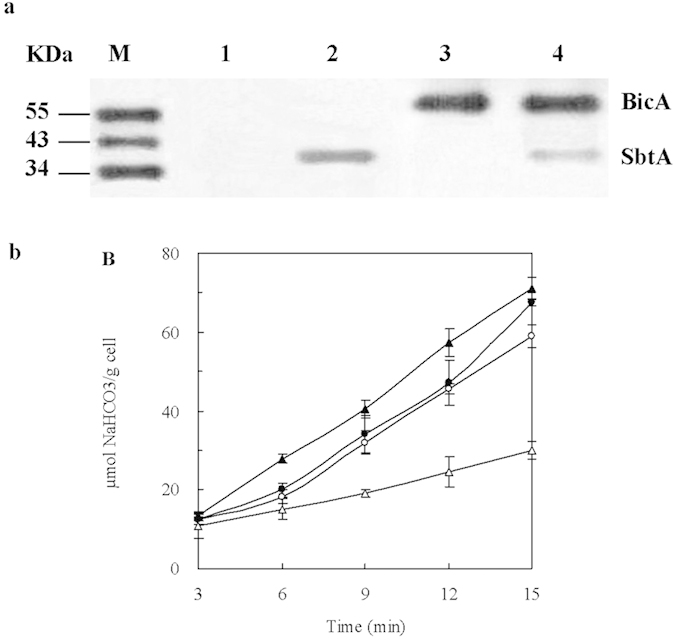
BicA and SbtA expression via His tag (**a**) and the uptake of HCO_3_^−^ in *E. coli* AFP111 (**b**). Overexpressed proteins were detected by western-blots. Lane 1: Tang1505 (pTrc99A), lane 2: Tang1506 (*sbtA*), lane 3: Tang1507 (*bicA*), lane 4: Tang1508 (*sbtA* and *bicA*). M corresponds to the molecular weight marker lanes. Symbols for *E. coli* strains: Tang1505 (pTrc99A) (open triangle, 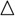), Tang1506 (*sbtA*) (black triangle, 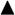), Tang1507 (*bicA*) (open circle, ○), Tang1508 (*sbtA* and *bicA*) (black circle, 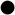).

In this work, a *trc* promoter was used to control the BicA and SbtA expression, so their expression levels were not affected by environmental factors, such as inorganic carbon species^19^,^29^, or light^30^,^31^. As shown in Fig. 2a, no BicA or SbtA expression was detected in Tang1505 (pTrc99A), while both BicA and SbtA were detected in Tang1508 (*sbtA* and *bicA*), indicating the feasibility of overexpression of these genes in *E. coli.* Overall, the expression of BicA was higher than that of SbtA. It was probably due to a wider codon adaptation and more stable mRNA of *bicA* (data not shown). From the transcription level, the expression of *bicA* was higher, correspondingly more BicA was synthetized. On the other hand, BicA is distinguishable as an extant member of the SulP family of anion transporters in eukaryotes and prokaryotes^32^,^33^. Some close homologs of BicA had been proved existing in several bacteria with high identity^19^. The reason why BicA could be better expressed in *E. coli* than SbtA, was probably because there is BicA homolog in *E. coli*.

As shown in Fig. 2b, HCO_3_^−^ transport activity was significantly improved in overexpression of *sbtA* (Tang1506), *bicA* (Tang1507) or both (Tang1508). After the expression of SbtA or BicA, the active transport system of HCO_3_^−^ was introduced into *E. coli* and the *E. coli* cells acquired the ability for active HCO_3_^−^ transportation. The highest transport flux of 71.08 μmol HCO_3_^−^ g^−1^ cell was obtained with Tang1506 (*sbtA*). It was 1.4-times higher than that of Tang1505 (pTrc99A). The HCO_3_^−^ uptake in cells overexpressing BicA was lower than that of SbtA expressing cells. It were different from previous reports. In cyanobacteria, BicA has a moderate photosynthetic uptake affinity for HCO_3_^−^ (K_0.5_ of ≈38 μM). It was able to support a high photosynthetic flux rate, while the SbtA transporter supported a low flux rate but with a high uptake affinity (K_0.5_ < 2 µM)^19^.

**Figure 3:**
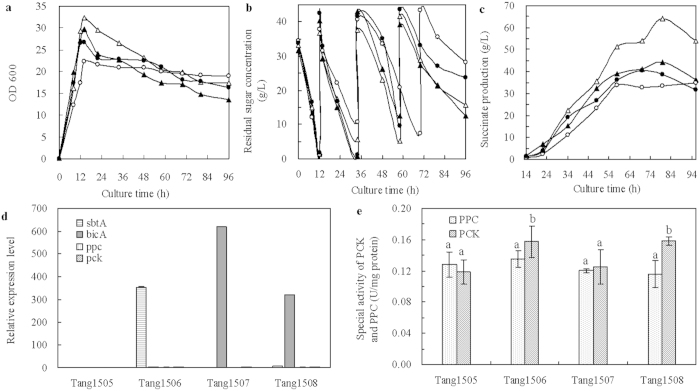
Time courses of dry cell weight (**a**), residual sugar concentration (**b**), the production of succinate (**c**), relative expression levels of *sbtA*, *bicA*, *ppc* and *pck* (**a**), and the specific activities of the PCK and PPC (**e**) under the expression of the HCO_3_^−^ transporters in fed-batch fermentation. T The symbols for *E. coli* strains are the same as those in Fig. 2. Error bars show standard deviation (n = 3). Different letters (e.g., (**a,b**)) were assigned to significantly different groups, and for the results between two groups.

As shown in Fig. 3a, overexpression of *bicA* and/or *sbtA* hinders cell growth, and the inhibitory effect of SbtA on cell growth was less than that caused by BicA. It probably due to the highly detrimental effect on the host cells caused by the overexpression of the membrane protein^34^. The expression of BicA was higher than that of SbtA. The increased expression of heterologous membrane proteins interferenced the cellular morphology and function. As a result, cell growth was negatively affected. The time profiles of glucose obtained by mutants were similar, except for Tang1507 (*bicA*) (Fig. 3b).

The effect of the expression of BicA and SbtA on succinate production was shown in Fig. 3c. It showed that overexpression of BicA or SbtA, or both had a negative effect on the succinate biosynthesis. One possible reason for this decrease was attributed to the negative effect associated with the high concentration of HCO_3_^−^ on the overall cell metabolism. BicA and SbtA are both Na^+^-dependent HCO_3_^−^ transporters^18^,^19^. Adequate Na^+^ levels were provided by the NaOH, which was used to control pH, to ensure steady expression of the transporters. HCO_3_^−^ accumulated in the cell, while CO_2_ fixation was not enhanced to effectively fix the intracellular HCO_3_^−^. Thus the original intracellular metabolic environment was disordered by the increased intracellular pH, which was caused by the increased intracellular HCO_3_^−^. This observation was supported by the slower cell growth (Fig. 3a).

As shown in Fig. 3d, when the two genes were expressed, the *sbtA* and *bicA* was up-regulated by 8- to 618-fold, respectively. And when *sbtA* was expressed, *pck* was up-regulated by 2.1-fold and 2.4-fold. Correspondingly, the enzyme activity of PCK in Tang1506 (*sbtA*) (0.16 U mg^−1^) or Tang1508 (*sbtA* and *bicA*) (0.16 U mg^−1^) was higher than that in Tang1505 (pTrc99A) (0.12 U mg^−1^) (Fig. 3e). This suggested that the PCK was activated by the expression of SbtA, but not BicA. No significant difference of PPC enzyme activity was found among the different strains (P > 0.05).

### Collaborative metabolic regulation of CO_2_ transport and fixation

#### Co-expression of CO_2_ transport and CO_2_ fixation genes

Succinate production involves two major steps: CO_2_ uptake and CO_2_ fixation. To achieve higher production of succinate, the two steps should be in succession, linked closely and complementing each other. To investigate whether the activation of CO_2_ transport and CO_2_ fixation had a synergistic effect in improving succinate production, co-expression of both genes was carried out by the combined expression of 1) two transport genes coupled with one fixation gene; 2) two fixation genes coupled with one transportation gene; and 3) two transport genes coupled with two fixation genes.

**Figure 4:**
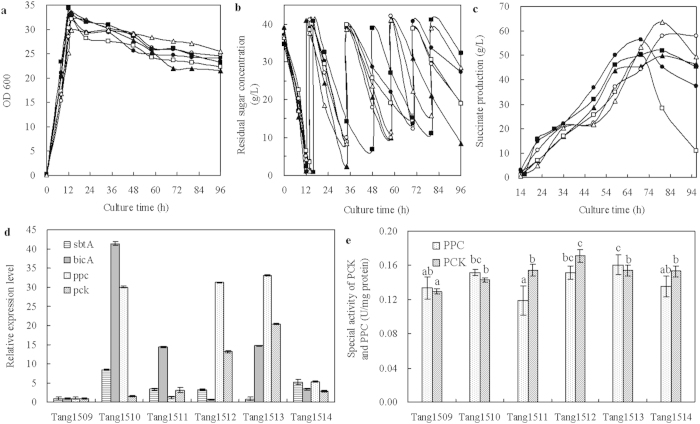
Time courses of dry cell weight (**a**), residual sugar concentration (**b**) and the production of succinate (**c**), relative expression levels of *sbtA*, *bicA*, *ppc* and *pck* (**d**), and the specific activities of the PCK and PPC (**e**) under the collaborative expression of multiple HCO_3_^−^ transporters and CO_2_ fixation genes in fed-batch fermentation. Symbols for *E. coli* strains: Tang1509 (pTrc99A and pACYC184) (open triangle, 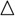), Tang1510 (*sbtA*, *bicA* and *ppc*) (black triangle, 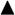), Tang1511 (*sbtA*, *bicA* and *pck*) (open circle, ○), Tang1512 (*sbtA*, *ppc* and *pck*) (black circle, 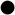), Tang1513 (*bicA*, *ppc* and *pck*) (open square, 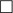), Tang1514 (*sbtA*, *bicA*, *ppc* and *pck*) (black square, 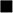). Different letters (e.g., (**a–c**)) were assigned to significantly different groups, and for the results between two groups, a combination of the two corresponding letters was used (e.g., **a,b** and **b,c**).

As shown in Fig. 4a, all strains showed similar rates of dry cell weight increase. The glucose consumption rates of Tang1512 (*sbtA*, *ppc* and *pck*) and Tang1513 (*bicA*, *ppc* and *pck*) were lower than that of Tang1509 (pTrc99A and pACYC184), Tang1510 (*sbtA*, *bicA* and *ppc*) or Tang1511 (*sbtA*, *bicA* and *pck*) (Fig. 4b). As shown in Fig. 4c, the highest succinate production (57.9 g L^−1^) among the strains that expressed any combination of genes was obtained from Tang1511 (*sbtA*, *bicA* and *pck*), which was still lower than that of Tang1509 (pTrc99A and pACYC184). Correspondingly, lower succinate productivity and succinate yield on dry cell weight (DCW) was also observed when multiple CO_2_ transport and fixation genes were overexpressed (Table 3). Compared with the succinate production obtained by CO_2_ transport overexpression (34.8–44.1 g L^−1^), when the CO_2_ transport and CO_2_ fixation genes were co-expressed, succinate production was improved (49.9–57.9 g L^−1^). It probably because HCO_3_^−^ transported into cells under the overexpression of transport proteins was promptly fixed. The metabolic disturbance caused by high concentration of intracellular HCO_3_^−^ was partially eliminated. However, the negative effect caused by membrane protein expression still exists. It suggested that the flux of transportation or fixation was still uncoordinated and unstable. It also suggests that a better coordinated regulation of CO_2_ transport and CO_2_ fixation is important in metabolism.

As shown in Fig. 4d, when the CO_2_ transport and fixation genes were individually or combinedly expressed, the *sbtA*, *bicA*, *ppc* or *pck* was up-regulated by more than 2-fold, correspondingly. The significant higher activity of PCK was obtained by recombined strains, and the overexpression of PPC and/or PCK showed insignificantly improved effect on the succinate biosynthesis (Fig. 4e).

#### Co-expression of single CO_2_ transport and fixation gene

**Figure 5:**
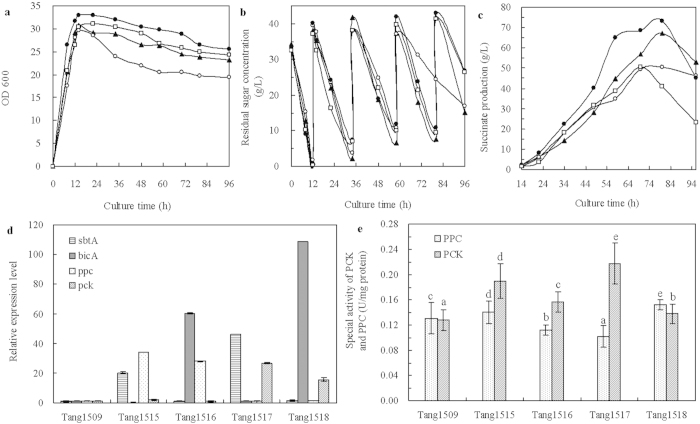
Time courses of dry cell weight (**a**), residual sugar concentration (**b**), the production of succinate (**c**), relative expression levels of *sbtA*, *bicA*, *ppc* and *pck* (**d**), and the specific activities of the PCK and PPC (**e**) under the collaborative expression of single HCO_3_^−^ transporter and single CO_2_ fixation gene in fed-batch fermentation. Symbols for *E. coli* strains: Tang1515 (*sbtA* and *ppc*) (black triangle, 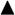), Tang1516 (*bicA* and *ppc*) (open circle, ○), Tang1517 (*sbtA* and *pck*) (black circle, 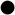), Tang1518 (*bicA* and *pck*) (open square, 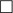). Error bars show standard deviation (n = 3). Different letters (e.g., (**a–e**) were assigned to significantly different groups, and for the results between two groups.

In order to find out the best combination of CO_2_ transport and CO_2_ fixation that has a synergistic effect in improving succinate production, we further investigated the expression of single transport gene coupled with single fixation gene. As shown in Fig. 5a, the biomass production was similar, except that Tang1517 (*sbtA* and *pck*) grew slightly better, which may be due to the increased HCO_3_^−^ supplement and increased PCK activity leading to more active cell metabolism. The higher PCK activity leads to more OAA and ATP formation, and the energy conserved by PCK was beneficial for cell growth. In addition, no significant difference was observed for glucose consumption among the four strains, Tang1515 (*sbtA* and *ppc*), Tang1516 (*bicA* and *ppc*), Tang1517 (*sbtA* and *pck*) and Tang1518 (*bicA* and *pck*) (Fig. 5b).

The succinate production was also greatly improved when a single transport and a single fixation gene were co-expressed (Fig. 5c). The highest succinate production was 73.4 g L^−1^ from Tang1517 (*sbtA* and *pck*), which was 13.3%, 66.4% and 15.0% higher than that obtained from Tang1503 (*pck*), Tang1506 (*sbtA*) and Tang1509 (pTrc99A and pACYC184), respectively. This result indicates that the best combination of transport and fixation genes was represented by *sbtA* and *pck*. HCO_3_^−^ transported into cells under the overexpression of SbtA was promptly fixed by PCK. Transport and fixation flux balanced. In addition, the succinate productivity, succinate yield on DCW and succinate yield on glucose obtained by Tang1517 attained the highest value (Table 3). When *bicA* was co-expressed with *ppc* (Tang1516) or *pck* (Tang1518), succinate production was lower than that obtained by Tang1509 (pTrc99A and pACYC184). However, compared with the succinate production obtained from Tang1507 (*bicA*) (Fig. 3c), the inhibitory effect on succinate biosynthesis caused by BicA alone was attenuated by combined expression with CO_2_ fixation gene. It suggested that the collaborative metabolic regulation was effective on improving the utilization rate of CO_2_.

As shown in Fig. 5d, when the CO_2_ transport and fixation gene were combinedly expressed, the *sbtA*, *bicA*, *ppc* or *pck* was up-regulated by more than 2-fold, correspondingly. The PCK activities of Tang1515 (*sbtA* and *ppc*), Tang1516 (*bicA* and *ppc*), Tang1517 (*sbtA* and *pck*) and Tang1518 (*bicA* and *pck*) were 0.19, 0.16, 0.22, and 0.14 U mg^−1^ protein, respectively (Fig. 5e). This result was positively correlated with the succinate biosynthesis (Fig. 5c). The corresponding PPC activities were 0.14, 0.10, 0.11, and 0.15 U mg^−1^ protein, respectively. When *sbtA* and *ppc* were expressed together, there was no obvious improvement in PPC activity probably due to various factors affecting the activity of PPC, such as aspartate and citrate^35^. On the other hand, PPC has a *K*_*m*_ for bicarbonate of 0.1 μM, whereas PCK has a *K*_*m*_ for bicarbonate of 13 μM^36^,^37^. PPC is more sensitive to the concentration of bicarbonate than PCK, and carries out PEP carboxylation at a lower concentration of HCO_3_^−^. As the active substrate for PPC, when the concentrations of intracellular HCO_3_^−^ was at a high level, PPC activity was likely limited owing to substrate inhibition.

PCK catalyzed the reaction at a higher concentration of HCO_3_^− 14^. As previously reported, when 20 g L^−1^ of NaHCO_3_ was added, succinic acid production in recombinant *E. coli* overexpressing PCK was 2.2-fold higher than that observed in the wild-type strain^38^. Interestingly, we noted that when SbtA was expressed, PCK was activated (Fig. 3d,e). It may be the reason why the higher activity of PCK reached the peak value when *sbtA* and *pck* were expressed together.

## Conclusions

To improve succinate production, two sets of genes, one for CO_2_ fixation (*ppc* and *pck*) and another for CO_2_ transport (*sbtA* and *bicA*), were overexpressed individually or in various combinations in *E. coli*. Our results showed that overexpression of either set of genes individually did not improve succinate production. To our surprise, when the two sets of genes (at least 3 genes) were co-expressed, no improvement on succinate production was observed. However, when only one gene from each gene set was co-expressed, succinate production was significantly increased, especially for gene combination of *pck* and *sbtA*, which reached the highest succinate production (73.4 g L^−1^) compared with other strains. Based on our results, we believe that collaborative regulation of CO_2_ transport and fixation is critical for succinate production. Imbalanced gene expression located upstream and downstream of the metabolic pathway may cause harmful effects to cell growth and succinate production.

## Additional Information

**How to cite this article**: Zhu, L.-W. *et al.* Collaborative regulation of CO_2_ transport and fixation during succinate production in *Escherichia coli*. *Sci. Rep.* **5**, 17321; doi: 10.1038/srep17321 (2015).

## Figures and Tables

**Table 1:** Strains and plasmids used in this study.

Strains	Relevant characteristics	Sources or reference
AFP111	F + λ- *rpo*S396(Am) rph-1 Δ*pflAB*::Cam *ldhA*::Kan *ptsG*	20
*Synechocystis* PCC6803	Providing *sbtA*, *bicA* and *ppc* gene	21
*Bacillus thuringiensis* BMB171	A crystalliferous *B. thuringiensis* mutant, providing *pck* gene	22
DH5α	F-φ80 lacZΔM15Δ(lacZYA-argF)U169 end A1 recA1 hsdR17(rk-,mk-) sup E44 λ-thi-1 gyrA96 relA1 phoA	TransGen Biotech
Tang1501	AFP111/pACYC184	This study
Tang1502	AFP111/pTrc-*ppc*	This study
Tang1503	AFP111/pACYC-*pck*	This study
Tang1504	AFP111/pTrc-*ppc* + pACYC-*pck*	This study
Tang1505	AFP111/pTrc99A	This study
Tang1506	AFP111/pTrc-*sbtA*	This study
Tang1507	AFP111/pTrc-*bicA*	This study
Tang1508	AFP111/pTrc-*bicA-sbtA*	This study
Tang1509	AFP111/pTrc99A + pACYC184	This study
Tang1510	AFP111/pTrc-*ppc*-*bicA-sbtA*	This study
Tang1511	AFP111/pTrc-*bicA-sbtA* + pACYC-*pck*	This study
Tang1512	AFP111/pTrc-*ppc*-*sbtA* + pACYC-*pck*	This study
Tang1513	AFP111/pTrc-*ppc*-*bicA* + pACYC-*pck*	This study
Tang1514	AFP111/pTrc-*ppc*-*bicA-sbtA* + pACYC-*pck*	This study
Tang1515	AFP111/pTrc-*ppc*-*sbtA*	This study
Tang1516	AFP111/pTrc-*ppc*-*bicA*	This study
Tang1517	AFP111/pTrc-*sbtA* + pACYC-*pck*	This study
Tang1518	AFP111/pTrc-*bicA* + pACYC-*pck*	This study
Plasmids		
pTrc99A	*Ap*^*R*^, pBR322 ori, *trc* promoter, *lacI*^*q*^	Invitrogen
pTrc-*sbtA*	pTrc99A with *sbtA* gene	This study
pTrc-*bicA*	pTrc99A with *bicA* gene	This study
pTrc-*bicA-sbtA*	pTrc99A with *sbtA* and *bicA* gene	This study
pTrc-*ppc*	pTrc99A with *ppc* gene	This study
pTrc-*ppc*-*sbtA*	pTrc99A with *ppc* and *sbtA* gene	This study
pTrc-*ppc*-*bicA*	pTrc99A with *ppc* and *bicA* gene	This study
pTrc-*ppc*-*bicA-sbtA*	pTrc99A with *ppc*, *sbtA* and *bicA* gene	This study
pACYC184	*cat*^*R*^, *tet*^*R*^, p15A ori	NEB
pACYC-*pck*	pACYC184 with *trc* promoter and *pck* gene	This study

**Table 2:** Primers used in this study^a^.

Primer sets	Relevant characteristics	Sources
SbtA-SacI-H	GACC***GAGCTC***ATGGATTTTTTGTCCAATTTCTTGACGGACTTCGTGGG	This study
SbtA-B-His	GAA***GGATCC***TTA**GTGATGGTGATGGTGATG**ACCTGCACCAAGGGTCTGGGC	This study
BicA-EcoRI-H:	GACG***GAATTC***ATGCAAATAACTAACAAAATTCATTTTAGGAACCTGCAGGGGGA	This study
BicA-B-His	GAAA***GGATCC***TTA**ATGGTGATGGTGATGGTG**GTATGTGGTCTGGACGGAAG	This study
P-trc-XbaI	GCC***TCTAGA***TGACAATTAATCATCCGGCTCGTATAATGTGTGG	This study
SbtA-SalI-His	GAC***GTCGAC***TTA**GTGATGGTGATGGTGATG**ACCTGCACCAAGGG	This study
*ppc*-EcoRI	CCG***GAATTC***GATATGAACTTGGCAGTTCCTGCATTCGG	This study
*ppc*-BamHI	ACC***GGATCC***TCAACCAGTATTACGCATTCCGGCCGC	This study
BicA-salI	G***GTCGAC***TTAATGGTGATGGTGATGGTGGTATGTGGTCTGG	This study
BamHI-SD-BicA	GACG***GGATCC**AGGAGG*ATGCAAATAACTAACAAAATTCATTTTAGGAACC	This study
BicA-XbaI	GATT***TCTAGA***TTAATGGTGATGGTGATGGTGGTATGTGGTCTGGACGGAAG	This study
*pck*-SacI	CGAGCTCATGCGAAATGAAGGGAAATT	This study
*pck*-HindIII	CCCAAGCTTTTAAGCGATTGGACCGCCTA	This study
trc*-pck*-DrdI	CGCGACATCGAAGTCGCAGGTCGTAAATCACTGC	This study
trc*-pck*-BclI	CGCTGATCATTAAGCGATTGGACCGCCTAAG	This study
SbtA-F(RT)	GCATGGCAATTCGGAACTCCAAC	This study
SbtA-R(RT)	CAGCCATTGTAGAGCCACTGACTG	This study
BicA-F(RT)	CAGGGCATCGGCAATGTAATGTC	This study
BicA-R(RT)	GAATGGTAGCCGCCAATTTAGCTG	This study
PCK-F(RT)	TTTGCAGGCGCTGACCGCAATTAC	This study
PCK-R(RT)	CAGCTGGATCAGCTTTGAAGTTCG	This study
PPC-F(RT)	AACCATTGCCAGTGGGCATTGAC	This study
PPC-R(RT)	CCGGATGGTGTGACGGACAATTTC	This study
16S rRNA-F	GCTAATACCGCATAACGTCGCAAG	This study
16S rRNA-R	GGACCGTGTCTCAGTTCCAGTGTG	This study

^a^Italic and bold bases encode restriction site and underlined bases encode 6 * His tag.

**Table 3:** Effect of collaborative regulation of CO_2_ transportation and fixation on the production of succinate.

Strains	Succinate productivity (g L^−1^ h^−1^)	Succinate yield on DCW (g g^−1^ DCW)^a^	Succinate yield on glucose (g g^−1^ glucose)
Tang1509 (pTrc99A and pACYC184)	0.96	4.82	0.54
Tang1510 (*sbtA*, *bicA* and *ppc*)	0.72	3.22	0.38
Tang1511 (*sbtA*, *bicA* and *pck*)	0.69	4.02	0.39
Tang1512 (*sbtA*, *ppc* and *pck*)	0.95	3.54	0.60
Tang1513 (*bicA*, *ppc* and *pck*)	0.88	3.49	0.57
Tang1514 (*sbtA*, *bicA, ppc* and *pck*)	0.79	3.34	0.53
Tang1515 (*sbtA* and *ppc*)	0.99	4.80	0.49
Tang1516 (*bicA* and *ppc*)	0.74	4.54	0.46
Tang1517 (*sbtA* and *pck*)	1.08	4.94	0.58
Tang1518 (*bicA* and *pck*)	0.87	3.58	0.42

^a^At an OD_600_ of 1.0, *E. coli* has a concentration of 0.44 g dry cell weight per liter.
